# Modeling Occurrence of Urban Mosquitos Based on Land Use Types and Meteorological Factors in Korea

**DOI:** 10.3390/ijerph121013131

**Published:** 2015-10-20

**Authors:** Yong-Su Kwon, Mi-Jung Bae, Namil Chung, Yeo-Rang Lee, Suntae Hwang, Sang-Ae Kim, Young Jean Choi, Young-Seuk Park

**Affiliations:** 1 Department of Life and Nanopharmaceutical Sciences, Kyung Hee University, Seoul 02447, Korea; E-Mails: davy3021@hanmail.net (Y.-S.K.); mjbae@nnibr.re.kr (M.-J.B.); 2 Department of Biology, Kyung Hee University, Seoul 02447, Korea; E-mails: namilchung@gmail.com (N.C.); lyrenter@nate.com (Y.-R.L.); 3 Freshwater Bioresources Research Division, Nakdonggang National Institute of Biological Resources, Sangju, Gyeongsanbuk-do 37242, Korea; 4 College of Electrical Engineering & Computer Science, Kookmin University, Seoul 02707, Korea; E-Mail: sthwang@kookmin.ac.kr; 5 Yeongdeungpo-gu Health Center, Yeongdeungpo-gu, Seoul 07260, Korea; E-Mail: daisy@ydp.go.kr; 6 WISE Institute, Hankuk University of Foreign Studies, Seoul 02450, Korea; E-Mail: junowise@hufs.ac.kr

**Keywords:** urban mosquito, land use type, meteorological factor, random forest

## Abstract

Mosquitoes are a public health concern because they are vectors of pathogen, which cause human-related diseases. It is well known that the occurrence of mosquitoes is highly influenced by meteorological conditions (e.g., temperature and precipitation) and land use, but there are insufficient studies quantifying their impacts. Therefore, three analytical methods were applied to determine the relationships between urban mosquito occurrence, land use type, and meteorological factors: cluster analysis based on land use types; principal component analysis (PCA) based on mosquito occurrence; and three prediction models, support vector machine (SVM), classification and regression tree (CART), and random forest (RF). We used mosquito data collected at 12 sites from 2011 to 2012. Mosquito abundance was highest from August to September in both years. The monitoring sites were differentiated into three clusters based on differences in land use type such as culture and sport areas, inland water, artificial grasslands, and traffic areas. These clusters were well reflected in PCA ordinations, indicating that mosquito occurrence was highly influenced by land use types. Lastly, the RF represented the highest predictive power for mosquito occurrence and temperature-related factors were the most influential. Our study will contribute to effective control and management of mosquito occurrences.

## 1. Introduction

Mosquitoes are one of the most notorious and influential insects in the public health field [1]. Even though only a few species among the 3000 mosquito species identified in the world are known for feeding on human blood, mosquitoes act as virus vectors for human-related diseases such as malaria, dengue fever, yellow fever, and West Nile virus, as well as animal diseases such as equine encephalitis and canine heartworm [2–6]. For example, *Culex pipiens* (*C. pipiens*) complex including *C. pipiens pallens* and *C. pipiens molestus*, which primarily occur in urban areas, are the primary vectors of West Nile virus [7], and *Aedes aegypti* (*A. aegypti*) is a key vector for the arboviruses causing dengue and yellow fever [8,9].

The occurrence and transmission of mosquito-borne diseases can be strongly related to the abundance of the host vector (*i.e.*, mosquito) [10,11]. Thus, for effective mosquito control, there have been several approaches related to quantifying and/or predicting the occurrence of mosquitoes within various spatial and temporal ranges. Udevitz *et al.* [12] predicted the occurrence of four mosquito species (*Anopheles punctipennis*, *Culex territans*, *Aedes atlanticus*, and *Psorophora ferox*) based on physico-chemical factors using stepwise logistic regression; Hales *et al.* [13] predicted the global distribution of dengue fever under current and future climates based on regressions with macroclimatic data; Peterson *et al.* [14] used a machine-learning approach to describe patterns of mosquito occurrence through space and time; Kearney *et al.* [15] predicted climate impacts on the potential range of the dengue fever vector, *A. aegypti*, based on biophysical models of energy and mass transfer; and Ruiz *et al*. [16] evaluated the impact of temperature and precipitation on West Nile virus infection in *Culex species* using random forest.

These approaches have also highlighted the roles and importance of meteorological factors, natural predators, competitors, prey, and mosquito density [17,18,19]. High tide frequency and low rainfall in the late dry season and early wet season led to higher population growth rates in *Aedes vigilax*, which facilitated mosquito breeding and subsequent abundance peaks, especially if low rainfall occurred during favorable tides [20]. Precipitation can support and maintain a “comfort zone” for mosquito egg laying as well as growth during the immature larval phase even though it can also be detrimental depending on the strength, intensity, and amount of precipitation [6,21,22]. Warm temperatures can trigger peak occurrences of mosquitoes [16,23,24,25,26,27], whereas winter freezing can cause high mortality of mosquito eggs, larvae, and adults [28].

Changes in land use (e.g., urban expansion, industrialization, *etc*.) have influenced the spread and dispersal of mosquitoes. For instance, increased soil moisture due to irrigation development and/or the construction of dams promoted a rapid density peak of *Culex quinquefasciatus* [29]. The continuous increase in artificial ecosystems as a result of industrialization and urbanization has enhanced successful introduction and establishment of vector mosquitoes. Generally, urban mosquitoes breed in open water habitats and stagnant water with high organic content such as sewage ditches, leading to successful establishment of mosquito populations [30,31,32,33,34,35,36].

However, to date, despite several studies on the relationships between environmental variables and mosquito occurrence [16,37], there are insufficient studies on various scaled environmental factors (e.g., meteorological and land use) and/or their combined influence on the prediction of occurrence patterns of mosquitoes in Korea. Thus, we analyzed the occurrence patterns of urban mosquitos based on meteorological and land use types. We characterized the environmental conditions that cause higher probabilities of mosquito occurrence. We also investigated the importance of meteorological factors in predicting the occurrence of mosquitoes according to the land use type. Our study can contribute to effective control and risk assessment of mosquitoes.

## 2. Materials and Methods

### 2.1. Mosquito Data

The data on mosquito abundance were obtained from a public health center in Yeongdeungpo-gu, Seoul, Korea. In total, the data were collected at 12 sites using digital mosquito monitoring systems (DMS, Environmental Technology & Development: E-TND) [38] from May 2011 to October 2012. The system attracted female mosquitoes by diffusion of CO_2_ during the night when mosquitoes are active (6:00 p.m.–7:00 a.m.), and then the number of mosquitoes attracted to the system were counted using an infrared ray LED sensor. After counting the attracted mosquitoes, the system sent the collected data to a data server by a CDMA module in real-time. The monitoring system showed high efficiency (R^2^ > 0.85) between manual observation and automatic observation [38,39]. During the data preprocessing procedure, we excluded extreme and unrealistic values due to mechanical errors in the mosquito monitoring system (daily maximum observed value in waterfront area: 2000 individuals/day; daily maximum observed value in non-waterfront area: 200 individuals/day), based on consultation with a mosquito expert in the study areas. Missing data (e.g., extreme data, unrealistic data, *etc.*) were calculated with average value of observations before and after a day, then daily monitoring data were summarized to weekly data at each site, and then used in further analyses.

### 2.2. Environmental Data

To clarify the relationships between land use types and the occurrence of urban mosquitoes, land use data are extracted from each monitoring site within a 250-m buffer on a digital map using ArcGIS 9.3 (ESRI, Redlands, CA; http://ww.esri.com). The main components of the land use types in our monitoring areas were residential areas (RESD), commercial areas (COMM), culture and sport areas (CULT), public facilities areas (PUBL), artificial grassland (GRAS), inland wetland (WETL), bare soil (BARE), inland water (WATE), industrial areas (INDU), and traffic areas (e.g., roadways) (TRAF) (Table 1). The land use data were obtained from the Ministry of Environment, Korea.

**Table 1:** Characteristics of land use types and meteorological factors at 12 monitoring sites. Land use data was extracted from each monitoring site within a 250-m buffer, and then the proportions (%) of land use were calculated.

Category	Variables	Abbreviation	Mean (±SD ^*^)
Land use types	Residential area (%)	RESD	28.4 (±23.8)
	Commercial area (%)	COMM	16.2 (±13.2)
	Culture and sport area (%)	CULT	7.9 (±13.5)
	Public facilities area (%)	PUBL	8.5 (±11.8)
	Artificial grassland (%)	GRAS	7.5 (±10.3)
	Inland wetland (%)	WETL	0.0 (±0.1)
	Bare soil (%)	BARE	2.6 (±4.1)
	Inland water (%)	WATE	5.0 (±6.4)
	Industrial area (%)	INDU	0.1 (±0.3)
	Traffic area (%)	TRAF	23.8 (±6.7)
Meteorological factors	Average daily temperature (°C)	TempAVE	21.4 (±4.7)
	Maximum daily temperature (°C)	TempMAX	25.6 (±4.6)
	Minimum daily temperature (°C)	TempMIN	17.7 (±5.2)
	Average daily wind speed (m/s)	WindAVE	1.4 (±0.7)
	Average daily precipitation (mm)	PrcpAVE	0.3 (±1.1)
	Average daily humidity (%)	HumiAVE	70.6 (±13.3)
	Total daily precipitation (mm)	PrcpTOT	5.7 (±22.6)

^*^ SD: standard deviation.

**Figure 1:**
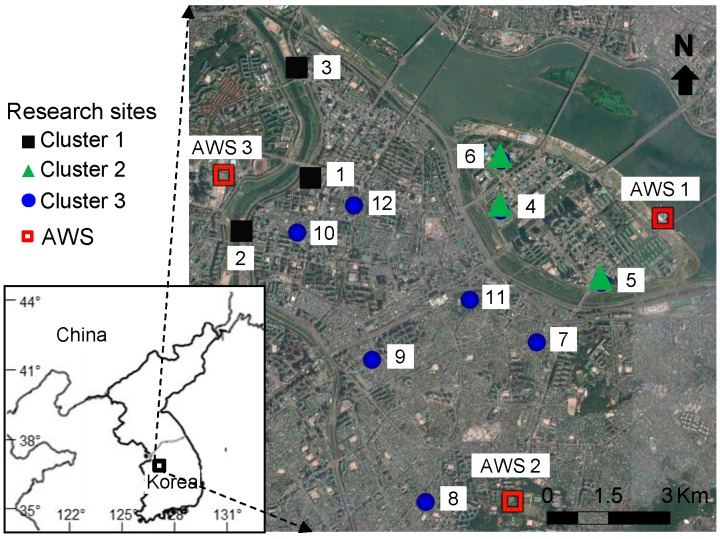
Location of sampling sites and automatic weather stations (AWS) in Yeongdeungpo-gu, Seoul, Korea.

In addition, we used seven meteorological factors, which can influence mosquito occurrence, including temperature (mean, maximum, and minimum), precipitation (average and total), wind speed, and humidity (Table 1). These factors were then applied to predict the degree of mosquito occurrence (see Data Analysis Section for the detailed explanations). All the meteorological data were obtained from three automatic weather stations (AWSs), which were located near the research sites and operated by the Korea Meteorological Administration (http://www.kma.go.kr). Three AWSs were located within a 2 km distance, and meteorological factor differences among the stations were small (Figure 1).

### 2.3. Data Analysis

#### 2.3.1. Classification of Habitats

We evaluated the relationships between the occurrence patterns of mosquitoes and land use types in two phases. First, a hierarchical cluster analysis was applied to classify the spatial difference in monitoring sites based on the proportions of land use types (Table 1) using Ward’s linkage method with a Euclidean distance measure. A multi-response permutation procedure (MRPP) [40] was applied to test whether or not there were significant differences among the clusters. Second, principal component analysis (PCA) was conducted based on mosquito abundance. Mosquito abundance at each site was transformed with natural log (ln (x + 1)). Then, the sites on the PCA ordination map were marked as groups from the cluster analysis extracted from the similarities of land use types. PCA is an indirect gradient analysis method for seeking the strongest linear correlation structure among variables [41], and it is a technique widely used for reducing the dimensions of multivariate problems. In PCA, eigenvalues, which explain a portion of the original total variance, are calculated. Each axis score using the eigenvector, which contains the coefficients of the linear equation for a given axis, was shown in an ordination [42]. Cluster analysis, MRPP, and PCA were performed using PC-ORD 5.0 [43].

#### 2.3.2. Prediction of Mosquito Occurrence

Mosquito abundance was categorized into four groups based on the boxplot (A: <25%, B: 25%–50%, C: 50%–75%, D: >75%) (Table 2). Based on meteorological factor differences, we predicted mosquito abundance categories using three machine learning techniques which have been widely applied in the prediction of various organisms: Support vector machine (SVM), classification and regression tree (CART), and random forest (RF) (e.g., [37]). Predictions were conducted separately in each land use type cluster defined by the cluster analysis. SVM is a machine learning system that uses a hypothesis space of linear functions in a high-dimensional space and is trained using a learning algorithm from optimization theory that implements learning bias derived from statistical learning theory [44]. In its classical implementation, it uses two classes (e.g., presence/absence) of training samples within a multidimensional feature space to fit an optimal separating hyperplane (in each dimension, the vector component is shown as a gray image) [45]. CART uses recursive partitioning to split the data into increasingly homogenous subsets, in terms of the dependent variable, to yield a binary decision tree, and the decision rules at the nodes use one or more of the independent variables [46]. The tree approach has several advantages over traditional classification methods. It is better suited to handling non-normal, non-homogeneous data sets [47]. The decision tree in CART consists of branching nodes, branches, and leaf nodes. The branching nodes represent the successive splits of the data set, each featuring the split variable and its split level. The branches indicate the path taken by individual cases, as determined by their value for the split variable. The leaf nodes display the resulting classification [48]. RF is a non-parametric method for predicting and assessing the relationships between a large number of potential predictor variables and response variables [49]. It is a model-averaging approach that generates hundreds or thousands of random trees built from a set of randomly selected predictors and observations [49,50]. After the trees have been built, data are entered into them and each grid square is classified by all trees. At the end of the run, the classification given by each tree is considered as a “vote”, and the classification of a grid square corresponds to the majority vote among all trees [49]. The RF model has demonstrated its learning and predicative power as well as its explanatory capacity by demonstrating a high capability for modeling ecological problems involving non-linear relationships between data [51]. After the learning process, accuracy test was applied to evaluate the predictability among three machine learning techniques. The accuracy (*i.e.*, the correct prediction rate) was computed based on the proportion (%) of the correct predictions between predicted and observed values [52].

The prediction models were conducted in the R computing environment (http://cran.r-project.org) with relating packages *e1071* [53] for SVM, *rpart* [54] for CART, and *CORElearn* [55] for RF. In each prediction model, the relative importance of meteorological factors for predicting mosquito occurrence was evaluated using the minimum description length (MDL), which measures the ability of an attribute to compress the data [56]. The values of MDL were rescaled to range from 0 to 100 to compare the relative importance of each environmental factor.

#### 2.3.3. Statistical Analysis

The differences in meteorological factors and land use types among clusters were compared using the Kruskal-Wallis test (K-W test). Dunn’s nonparametric multiple comparison tests were then used only if there were significant differences between clusters. The analyses were conducted using the statistical software STATISTICA 7.0 [57].

**Table 2:** Average abundance (per week) of mosquitoes at three different clusters. Abundance categories were defined based on the boxplot method and clusters were extracted from the Ward’s linkage method with Euclidean distance measures based on land use types.

Abundance Category		Cluster	
	1	2	3
A (<25%)	≤21.6	≤1.0	≤1.8
B (25–50%)	21.6–63.3	1.0–2.9	1.8–6
C (50–75%)	63.3–221.4	2.9–8.9	6–15.8
D (>75%)	>221.4	>8.93	>15.8

## 3. Results

### 3.1. Mosquito Occurrence

**Figure 2:**
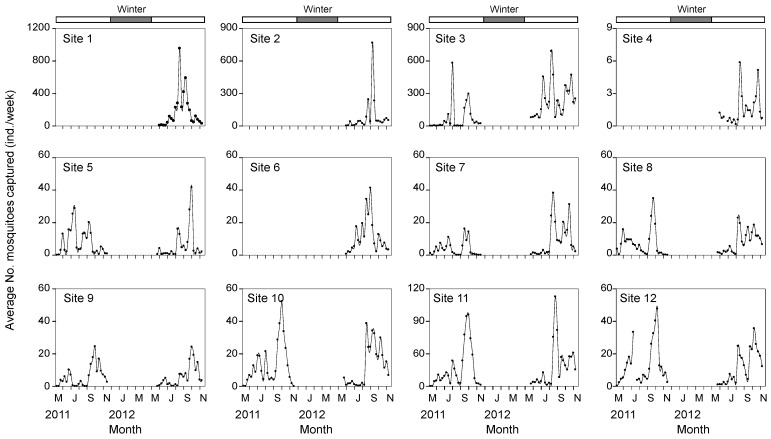
Changes in mosquito abundance at 12 monitoring sites. Sites 1, 2, 4, and 6 were only collected in 2012.

Mosquito abundance was highest in August and September in both years (Figure 2), showing relatively higher values in 2012. The highest abundance was observed at Sites 1, 2, and 3 (Site 1: 177.1 ± 228.5 (mean ± SD) individuals/week, Site 2: 86.1 ± 164.8 individuals/week, and Site 3: 149.9 ± 168.4 individuals/week). On the other hand, the abundance at Site 4 was much lower, ranging from one to six individuals/week during the monitoring period.

**Figure 3:**
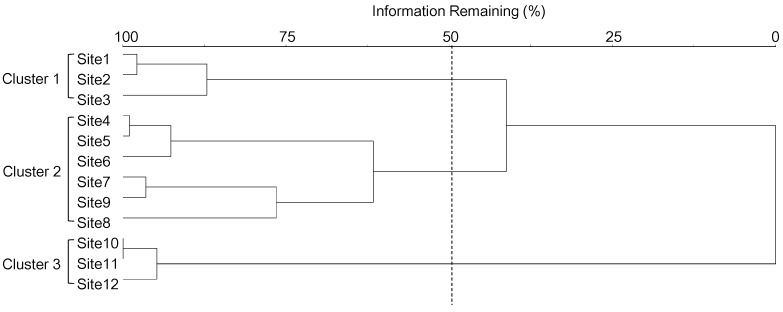
Classification of 12 monitoring sites based on the composition of land use types through a hierarchical cluster analysis with the Ward’s linkage method using the Euclidean distance measures.

### 3.2. Relationships between Mosquito Abundance and Land Use Types

**Figure 4:**
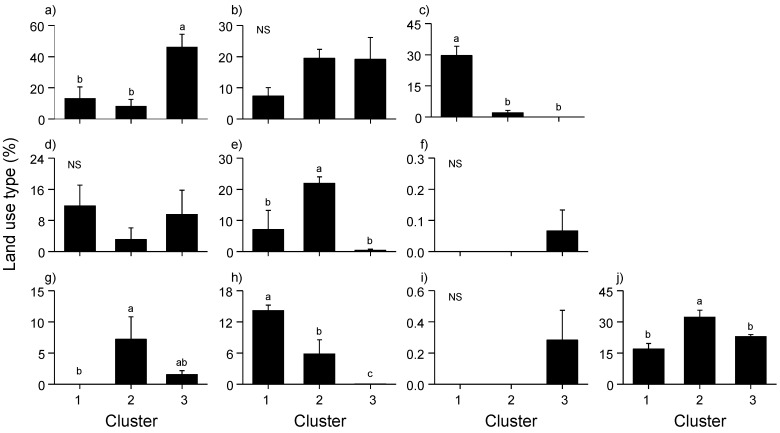
Differences in land use types among three different clusters. Land use types: (**a**) residential area (RESD), (**b**) commercial area (COMM), (**c**) culture and sport area (CULT), (**d**) public facilities area (PUBL), (**e**) artificial grassland (GRAS), (**f**) inland wetland (WETL), (**g**) bare soil (BARE), (**h**) inland water (WATE), (**i**) industrial area (INDU), and (j) traffic area (TRAF). Different alphabet letters in the figure represent significant differences based on Dunn’s multiple comparison tests (*P* < 0.05). NS indicates “not significantly different among clusters”.

**Figure 5:**
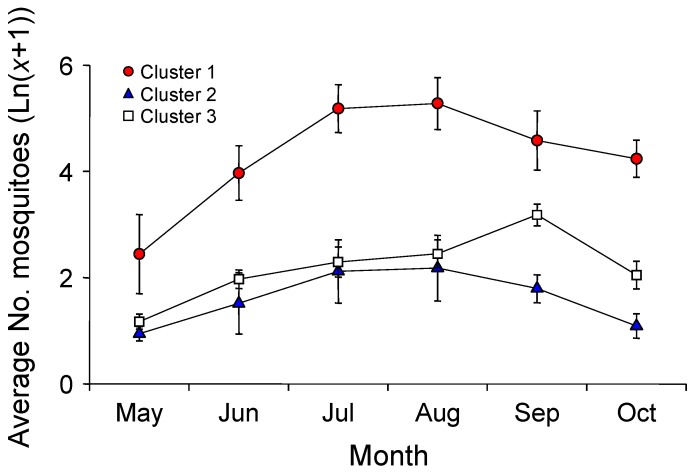
Changes in mosquito occurrence in each month from May to October in each cluster.

Twelve monitoring sites were classified into three clusters (1–3) based on the differences in land use composition in the surrounding area (Figure 3), and the MRPP showed significant differences among clusters (A = 0.32, *P* < 0.01). Cluster 1 showed the highest values for culture and sport area (29.57 ± 7.88%; mean ± SD) and inland water (14.13 ± 1.89%) land use types, whereas these values were the lowest in Cluster 3 (Dunn’s test, *P* < 0.05) (Figure 4). In Cluster 2, the percentage of artificial grassland (21.90 ± 3.56%), bare soil (7.20 ± 6.26%), and traffic area (32.30 ± 5.72%) were significantly higher than that in other clusters (Dunn’s test, *P* < 0.05). Residential area was highest in Cluster 3 (46.2 ± 20.0%) (Dunn’s test, *P* < 0.05). Mosquito abundance in each month (from May to October) was significantly different among the three clusters (K-W test, *P* < 0.05) (Figure 5). Mosquito abundance was significantly higher in Cluster 1 during all the sampling months.

**Figure 6:**
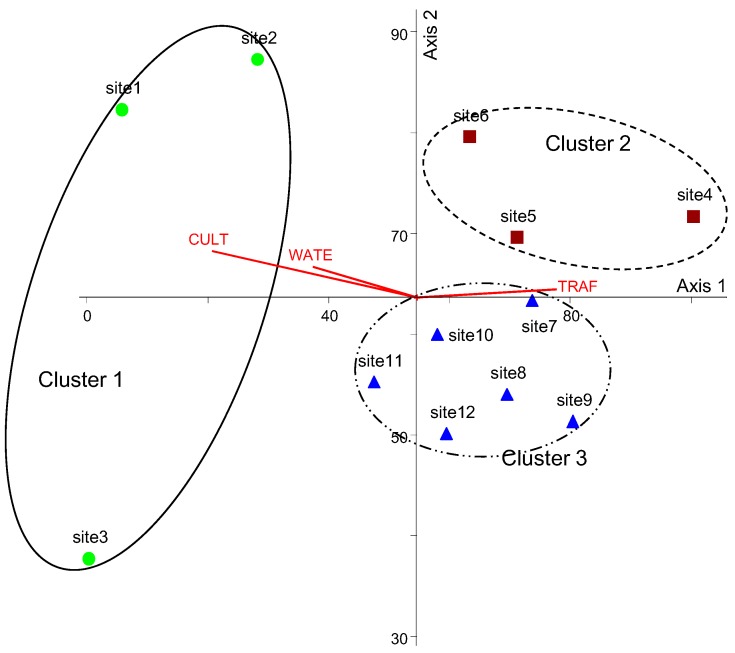
PCA ordination based on the differences in mosquito abundance. The land use types that showed significant correlation coefficients (*P* < 0.05) with the first two principal axes are shown as straight lines. The line length indicates the magnitude of the correlation and the line direction implies a negative or positive correlation with each axis. Land use types: culture and sport area (CULT); inland water (WATE); and traffic area (TRAF).

The mosquito occurrence differences according to the land use type were also reflected in PCA based on the mosquito abundance (cumulative variance of axes 1 and 2: 87.2%) (Figure 6). For instance, the monitoring sites in Cluster 1 were ordinated mainly on the left part of the ordination; sites in Cluster 2 were on the upper right part, and sites in Cluster 3 were on the right lower part. Considering the correlation between PCA axes and the relative ratio of land use types, culture and sport area (r = −0.82, *P* < 0.05) and inland water (r = −0.55, *P* < 0.05) were negatively correlated with axis 1, whereas traffic area (r = 0.68, *P* < 0.05) was positively correlated with axis 1.

### 3.3. Prediction of Mosquito Occurrence

**Figure 7:**
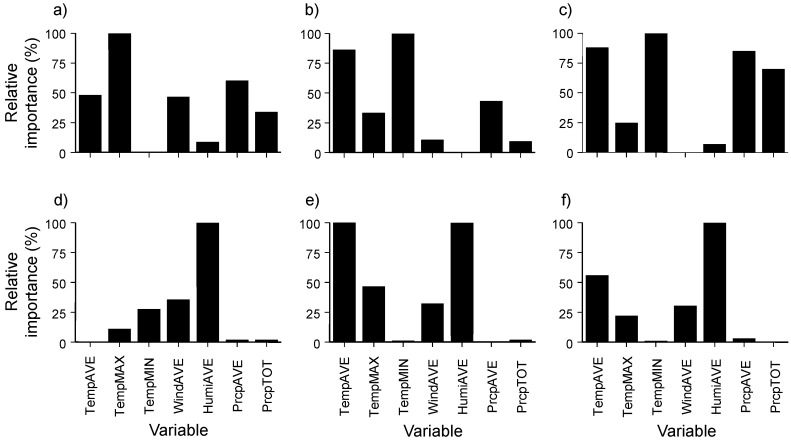
Relative importance of meteorological factors for the prediction of the category (categories) of mosquito abundance in each cluster using the random forest model: (**a**–**c**) prediction for the four categories of mosquito abundance; (**d**–**f**) prediction for the category of the highest abundance; and (**a**,**d**): Cluster 1, (**b**,**e**) Cluster 2, and (**c**,**f**) Cluster 3. Meteorological factors: average daily temperature (TempAVE; °C), maximum daily temperature (TempMax; °C), minimum daily temperature (TempMIN; °C), average daily wind speed (WindAVE; m/s), average daily humidity (HumiAVE; %), average daily precipitation (PrcpAVE; mm), and total daily precipitation (PrcpTOT; mm).

In the prediction of the four categories of mosquito abundance calculated from the box plot method, RF showed the highest predictive power (accuracy in Cluster 1: 0.80, Cluster 2: 0.71, and Cluster 3: 0.71) (Table 3), whereas SVM had the lowest predictive power (accuracy in Cluster 1: 0.59, Cluster 2: 0.61, and Cluster 3: 0.51). In the evaluation of the relative importance of environmental variables for the prediction of the four categories, mainly temperature-related factors showed high contributions based on the MDL in the RF model (Figure 7). For instance, minimum daily temperature was the most important variable for the prediction of mosquito abundance in Clusters 2 and 3, whereas maximum daily temperature was the most influential variable in Cluster 1.

In addition, when only the highest abundance group was predicted, the RF model showed the highest prediction power regardless of clusters (*i.e.*, accuracy in Cluster 1: 0.86, Cluster 2: 0.88, and Cluster 3: 0.88) (Table 3). Average humidity was the most influential variable for predicting the group with the highest mosquito abundance (Figure 6).

**Table 3:** Prediction accuracy of mosquito occurrences using three different models: support vector machine (SVM), classification and regression tree (CART), and random forest (RF). Categories (A–D) of mosquito abundance are given in Table 2.

Dataset	Model		Cluster	
		1	2	3
Four categories of abundance (A–D)	SVM	0.59	0.61	0.52
	CT	0.61	0.67	0.67
	RF	0.8	0.71	0.71
Category with highest abundance (D)	RF	0.86	0.88	0.88

## 4. Discussion

We analyzed the spatial and temporal differences in urban mosquito occurrence and the relationships with meteorological and habitat conditions such as land use type. Some mosquito habitats such as *Culex* spp. are easily found in urban environments, and mosquitoes often emerge in huge numbers from sewage and organic enriched water bodies [58]. In this study, we extracted the land use type characteristics at 12 monitoring sites using GIS, in order to examine the differences in habitat conditions around the monitoring sites. Twelve monitoring sites were classified into three clusters based on the land use type composition, which explained the strong relationship between land use types and mosquito occurrence. For instance, three monitoring sites (Sites 1–3) in Cluster 1 had relatively high mosquito abundance, and they were located near the Anyang stream with significantly higher proportions of inland water and culture and sport areas. Mosquitoes such as *Culex* spp. prefer unsanitary conditions and breeding in stagnant water with high organic content (e.g., sewage ditches and pools of water) [34,35,59]. Therefore, these sites had suitable habitats (Sites 1 and 2, ponds; and Site 3, storm-water pumping station) for mosquitoes. The oviposition behavior of mosquitoes is also attracted to water with high levels of organic substrates [31]. Meanwhile, six monitoring sites (Sites 7–12) in Cluster 3, which were in a residential area with a low proportion of inland water, displayed relatively low mosquito abundance. This indicates that the environmental conditions of these sites are not favorable for mosquitoes, although there are potential habitats for mosquitoes such as apartments and septic tanks in the residential area [60]. The low abundance might also be caused by mosquito control activities of the local government. Meanwhile, Cluster 2 showed significantly lower mosquito occurrence, although the monitoring sites have high proportions of grassland areas and intermediate proportions of inland water. Mercer *et al.* [61] reported that large open water bodies or running water are not suitable habitats because of the low nutrient concentrations and high predator abundance.

The multivariate techniques used in this study, including cluster analysis for classification and PCA for ordination, were useful for characterizing differences in mosquito abundance in different habitats over time. Twelve monitoring sites were separated by the first PCA axis, which explained 78.5% of the variance, reflecting differences in mosquito occurrences. For example, Cluster 1 with high mosquito abundance was located on the left side of axis 1, while other sites in Clusters 2 and 3 with low mosquito abundance were on the right part of axis 1. In particular, Site 4, located on the rightmost part of axis 1, showed the lowest abundance among the 12 monitoring sites. Meanwhile, the second axis reflected temporal changes in mosquito occurrence, although axis 2 exhibited only explained 8.7% of the variance. Sites 1 and 2, which showed a similar temporal pattern, were located in the upper part of axis 2, while Site 3, with relatively high abundance in September and October, was located on the lower part of axis 2. These occurrence results could also be influenced by meteorological conditions. For example, mosquito occurrence was suppressed by falling temperatures with losses of eggs and larvae by heavy rains during the monsoon [26,27]. Similar results have been reported for the seasonal change in mosquito oviposition activity in Shanghai, China [62] and Jeju, Korea [63].

Among the three prediction models in this study, the RF showed the highest prediction power for mosquito occurrence in urban areas. Similarly, [64] reported that the RF was the best-suited model for defining environmental conditions for several mosquito species, such as *Culiseta annulata*, *Anopheles claviger*, and *Ochlerotatus punctor*. Similar results have been documented in ecological studies comparing the performance of several prediction models [65,66]. As a prediction model, the RF has several advantages over other statistical methods, such as high classification accuracy, a novel method of determining variable importance, and the ability to model complex interactions among predictor variables [51]. Therefore, the RF offers powerful alternatives to traditional parametric and semiparametric statistical methods for the analysis of ecological data.

The emergence, dispersal, and feeding activity of urban mosquitoes were known to be highly related to meteorological factors, including temperature, precipitation, and humidity [59,67]. In our study, temperature-related factors such as average daily temperature and minimum and maximum daily temperature showed relatively higher importance in the prediction of the four categories of mosquito abundance, although there were some differences between clusters. Similarly, Vinogradova [68] emphasized the important role of temperature on the occurrence of *Culex pipiens pallens*. Increases in temperature facilitated rapid increase in mosquito populations [69]. The oviposition activity of mosquitoes (e.g., *Culex pipiens pallens*) also strongly depended on seasonal changes in temperature [62,70]. In addition, precipitation is particularly important for mosquito oviposition [6,71,72]. In our study, precipitation was important in the prediction of the four categories of mosquito abundance, as mosquitoes require water for the larval and pupal stages. Meanwhile, humidity was important for predicting the highest abundance category. High humidity can increase mosquito survival [73], and relative humidity influences longevity, mating, dispersal, feeding behavior, and oviposition of mosquitoes [74]. Bi *et al.* [75] reported that mosquitoes generally survive for a longer period of time and disperse more at higher humidity. However, extremely high humidity might indicate incoming rainfall, and therefore limit the dispersal of adult mosquitoes [71], and heavy rainfall might destroy the immature stages [67].

## 5. Conclusions

We analyzed the occurrence of urban mosquitoes according to differences in meteorological and habitat conditions. To evaluate the relationship between mosquito occurrence and habitat condition, combined multivariate analyses with cluster analysis and PCA were conducted. The monitoring sites were classified into three groups based on similarities in land use types, reflecting differences in mosquito occurrence in different land use types. Mosquito abundance was the highest in culture and sport areas and inland water land use area. The RF showed the highest prediction power for mosquito occurrence. The sensitivity analysis of the model showed that temperature-related factors such as average daily temperature, minimum and maximum daily temperature, and precipitation were highly important in the prediction of the four categories of mosquito abundance, whereas humidity was important for predicting high mosquito abundance. Our research can be applied for the efficient control of urban mosquitoes.
